# The National After-School Athletics Program Participation as a Tool to Reduce the Risk of Obesity in Adolescents after One Year of Intervention: A Nationwide Study

**DOI:** 10.3390/ijerph16030405

**Published:** 2019-01-31

**Authors:** Dominika Głąbska, Dominika Guzek, Blanka Mellová, Katarzyna Zadka, Katarzyna Żywczyk, Krystyna Gutkowska

**Affiliations:** 1 Department of Dietetics, Faculty of Human Nutrition and Consumer Sciences, Warsaw University of Life Sciences (SGGW-WULS), 159C Nowoursynowska Street, 02-776 Warsaw, Poland; katarzyna_zadka@sggw.pl; 2 Department of Organization and Consumption Economics, Faculty of Human Nutrition and Consumer Sciences, Warsaw University of Life Sciences (SGGW-WULS), 159C Nowoursynowska Street, 02-776 Warsaw, Poland; dominika_guzek@sggw.pl (D.G.); krystyna_gutkowska@sggw.pl (K.G.); 3 Nutrition, Health and Wellness Unit, Nestlé Polska S.A., 32 Domaniewska Street, 02-672 Warsaw, Poland; blanka.mellova@pl.nestle.com (B.M.); katarzyna.zywczyk@pl.nestle.com (K.Ż.)

**Keywords:** body mass, waist circumference, body composition, fat mass, muscle mass, physical activity, adolescents, #goathletics Study

## Abstract

Regular exercise during school hours is encouraged; however many children and adolescents fail to meet the recommendations during this time. Extracurricular activities may be a more appealing way for youth to achieve guidelines, and it is recommended that they attend two sessions each week. The aim of the study was to assess the influence of participation in a national physical activity program accompanied by nutritional education for trainers on the risk of obesity and body composition in a nationwide sample of boys and girls, after one year of intervention. The #goathletics Study was conducted in a group of 1014 adolescents aged 12–13: 507 individuals for the *Athletics for All* program (210 boys, 297 girls) and 507 pair-matched individuals not participating in any physical activity program (matching including: gender, age, city of residence). The body mass (kg), Body Mass Index (BMI) (kg/m^2^), waist circumference (WC) (cm), waist-to-height ratio (WHtR) (-) and body composition (%) (measured using bioelectrical impedance method) were compared in a gender-related sub-groups using *t*-Student test (for parametric distributions) or Mann-Whitney U test (for nonparametric distributions) and chi^2^ test (for the share of sub-groups). After one year of intervention, lower body mass percentile, BMI percentile, WC, WHtR and fat mass share, higher muscle mass share, as well as lower frequency of overweight/obesity and abdominal fat distribution were observed both for boys and girls participating in the physical activity intervention compared to the pair-matched controls. The after-school physical activity program accompanied by nutritional education for trainers may be a highly effective method for reducing the risk of obesity both for boys and girls, as regular participation is ensured.

## 1. Introduction

School-based physical activity interventions are indicated as a valuable approach to improve the physical performance [1] and general well-being [2] of children and adolescents, as well as to increase their physical activity enjoyment [3]. However, in a number of studies, the influence of school-based physical activity interventions on the body mass of children is not stated [4,5,6]. Even though an effect on anthropometric measurements is observed in some studies [7,8,9], it is not always noted. Moreover, in the meta-analysis of randomized trials by Guerra et al. [10], as well as in the meta-analysis by Harris et al. [11], it was observed that school-based interventions had no influence on either body mass or Body Mass Index (BMI) of children and adolescents.

The above-indicated insufficient effect of school-based programs on body mass of children and adolescents may result from their general reluctance to participate in, and avoidance of, school-based physical activity [12]. However, there is also a possibility to implement extracurricular physical activity interventions for children and adolescents that may be superior due to better adherence. In spite of the rather small number of studies analyzing the after-school physical activity interventions published thus far, such a possibility seems to be promising. In the cluster randomized trial of Martínez Vizcaíno et al. [13], it was stated that for primary school children, participation in such programs for a half year may lead to a reduction in adiposity (defined as excessive fat accumulation in adipose tissue) in boys and girls, measured as skin-fold thickness, as well as in girls, measured as a body fat share. Similarly, in a further study of Martínez Vizcaíno et al. [14], it was stated that a reduction of a waist circumference (WC) was also observed for boys and girls, but the prevalence of excessive body mass was not modified by the intervention. The similar result was also observed in the study of Yin et al. [15], in which a reduction in adiposity measured as a body fat share was found after three years of after-school intervention in primary school children. Also, in the study of Salcedo Aguilar et al. [16], it was observed that participation in an extracurricular program for two years led to decrease in the overweight frequency in primary school girls, but not boys. Moreover, in a systematic review and through meta-analysis of quasi-experimental, pilot, non-randomised or randomised trials of Mears & Jago [17], it was concluded that there is mixed evidence for increasing the physical activity levels by extracurricular physical activity interventions, and the stronger evidence was observed for overweight or obese children than for children with normal body weight. This differentiation between normal and excessive body weight adolescents is crucial, as for a normal body weight ones, the body mass reduction is not advisable, while for excessive body weight ones, the body mass reduction resulting from adiposity reduction should be obtained.

Regarding after-school physical activity programs, it must be emphasized that the studies conducted thus far have assessed various types and methodologies of interventions which influenced the observed results. The review of reviews by Demetriou et al. [18] indicated that a higher effectiveness is stated for programs conducted in school settings than for those conducted in community settings, for programs including two or more sessions a week than for those including less number of sessions, and for programs guaranteeing regular participation than for those not ensuring it. Moreover, the indicated review confirmed the observations of Salcedo Aguilar et al. [16]; that is, better results are observed for girls than boys when the intervention is associated with body weight control.

At the same time, in order to increase the effectiveness of the programs, it was proven to be more effective to include in educational programs not only physical activity, but also nutritional elements [19]. Such an approach is in accordance with the general strategy of the World Health Organization (WHO), indicating, as a major aim to achieve, the necessity of effective health programs conducted in school settings combining physical activity and dietary intervention [20]. Due to the fact that the main factor contributing to excessive body mass is an energy intake that exceeds energy expenditure [21], both actions, i.e., decreasing energy intake and increasing energy expenditure, may be effective. In spite of the fact that in some studies, physical activity is stated to be a more important determinant of the body mass of adolescents than dietary behaviors [22], the role of both is emphasized, referring to them as the “Big Two” [23].

As the influence of trainers, as prominent adult social leaders, is observed to cause positive changes to the eating behaviors of children [24], the other concept is to not influence participants directly, but to educate trainers to influence participants indirectly. Taking into account the promising role of extracurricular physical activity interventions, the aim of the present study was to assess the influence of participation in a national after-school athletics program (conducted in school settings, three sessions a week, ensuring regular participation and including nutritional education of trainers) on the risk of obesity and body composition in a nationwide sample of boys and girls after one year of intervention.

## 2. Materials and Methods

### 2.1. Ethics Statement

The #goathletics Study was conducted at the Department of Dietetics, Warsaw University of Life Sciences (WULS-SGGW). It was conducted according to the guidelines laid down in the Declaration of Helsinki. All procedures involving human subjects were approved by the Ethics Committee of the Faculty of Human Nutrition and Consumer Sciences of the Warsaw University of Life Sciences in Warsaw, Poland (No. 16/2017; 19.06.2017). Written informed consent was provided by all participants and their parents or legal guardians.

### 2.2. Intervention

The #goathletics Study was conducted in two groups of adolescents, aged 12–13. The study group included 507 individuals representative of *Athletics for All* (in Polish Lekkoatletyka Dla Każdego—LDK), which is a nationwide after-school physical activity program, and the control group included 507 pair-matched individuals recruited from a general population but not participating in any physical activity program.

The *Athletics for All* program (http://www.lekkoatletykadlakazdego.pl/), an after-school physical activity program which is free of charge, has been conducted in Poland since 2014. It is organized by the Polish Athletic Association and supported by the Ministry of Sport and Tourism and Nestlé Polska S.A. It is a real life intervention already implemented in all the regions of Poland, both in big cities and small towns in 807 training groups. Since 2014, over 300,000 children and adolescents from 1244 primary and secondary schools have participated in the program. The program was implemented with the aim of increasing physical activity in a group of primary school children and adolescents by providing regular athletics training.

In the current school year, in all the regions of Poland, there are 180,000 participants of the program (majority of them in the youngest age groups); almost 8000 primary school adolescents aged 12–13 participate. The regular attendance in the school year is ensured due to the fact that the program is free of charge and regular participation is a necessary condition (it is controlled for each session and recorded by trainer using a dedicated electronic application). Such an obligation guarantees the low dropout level that, in the group of adolescents aged 12–13, is currently 10% on an annual basis (based on the data for 2017/2018 school year).

As a part of the program, physical activity education session and athletics training are conducted in schools in various locations of Poland, while for a group of participating adolescents, three sessions a week are conducted (each session of a 90 min of moderate to high intensity physical activity) in which regular participation is required. Each trainer receives the identical outlines of the trainings with the specified aim of each training, necessary equipment and detailed instructions for all the elements of training. All the trainings are planned by the trainers from the Polish Athletic Association and are aimed at creating necessary abilities and skills for the athletics disciplines.

An additional nutritional education session was conducted for trainers at the beginning of the *Athletics for All* program, and it is repeated each year; during this session, additional educational materials are provided, and trainers are obliged to teach their participants not only about physical activity but also about proper balanced diet.

The additional element of the *Athletics for All* program that increased its attractiveness are the athletics events organized exclusively for its participants. In such events, including regular school cups, regional cups and a national cup, professional athletes and even star athletes (national and international champions) take part. So far, 2400 school cups and 300 regional cups have been organized, while the national cup is organized each year. Star athletes have contact with the *Athletics for All* program participants, meeting them, training together, and contacting them via a dedicated social network.

### 2.3. Study Groups and Design

#### 2.3.1. Participants

The #goathletics Study aimed to evaluate the effectiveness of the Athletics for All program, not only by the assessment of anthropometric measurements, but also by the assessment of physical performance and nutritional behavior [25]. For this study, the study groups were recruited following a two-stage recruitment procedure, in which adolescents aged 12–13 from all the regions of Poland (central, north, north-west, south-west, south, east) were included.

For the study group, the first stage of the recruitment procedure involved a random selection of primary schools in which the *Athletics for All* after-school physical activity program is being conducted. The geographical distribution of schools was based on Polish statistical data, and both big cities and small towns were included in the selection (random purposive sampling).

**Figure 1:**
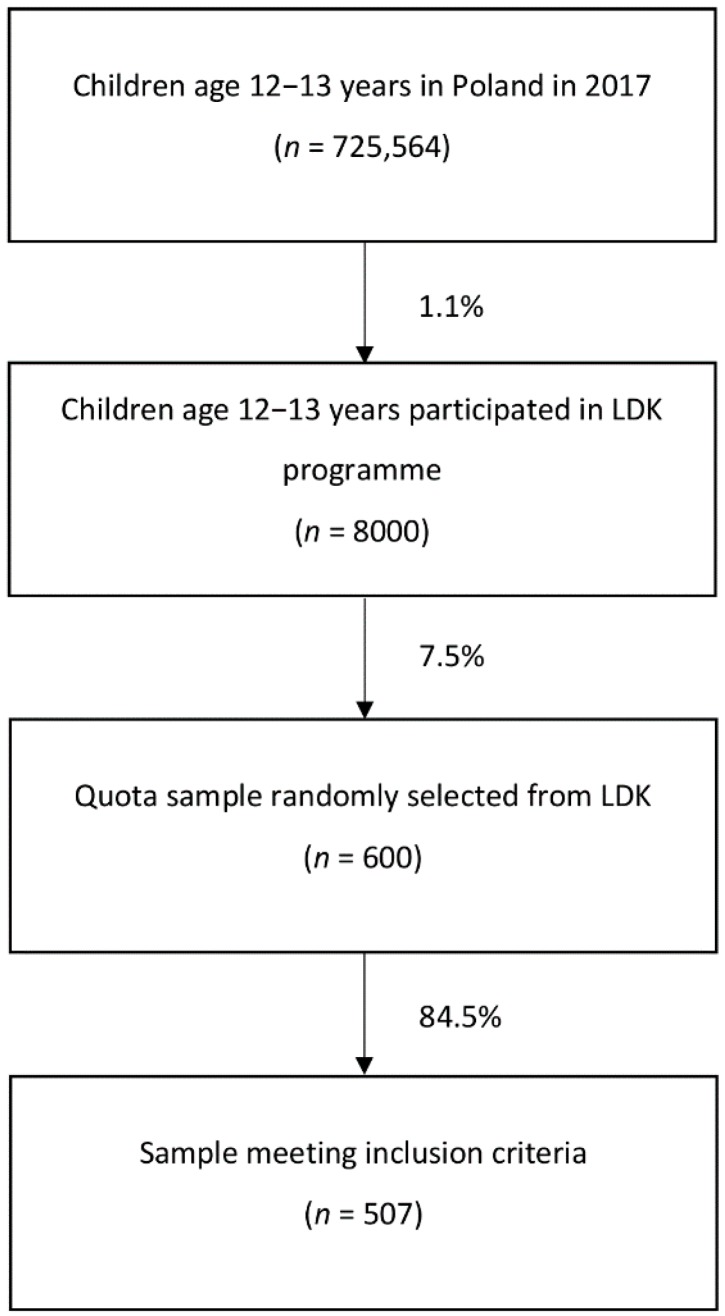
The selection of participants for the *Athletics for All* program, based on Statistical Demography Yearbook for 2017 [26].

The second stage of recruitment of the study group involved a random selection of participants in the *Athletics for All* program from each school (*n* = 600). The proportion of boys and girls selected was required to be in accordance with the general proportion of participants of the *Athletics for All* program (with a higher share of girls) (random sampling). Afterwards, the individuals meeting the inclusion criteria (Figure 1) were pair-matched with the control ones.

For the control group, the first stage of the recruitment procedure involved the selection of primary schools in which *Athletics for All* after-school physical activity program was not being conducted. The schools were required to be located in the same cities as schools chosen to recruit the study group, and so both big cities and small towns were included (purposive sampling).

The second stage of recruitment of the control group involved a random selection of adolescents who were neither participants in the *Athletics for All* program nor participants of any other physical activity program. The pair-matching procedure was applied, and the variables used for matching included gender, age, and city of residence. During this stage, the individuals eligible for recruitment, after being pair-matched with study individuals, were first identified, and then for each study individual, one pair-matched control individual was randomly chosen (pair-matched random sampling).

The inclusion criteria for the studied group were as follows:

- Caucasian,
- adolescents aged 12–13,
- participating for at least 1 year in the *Athletics for All* program training sessions regularly (three sessions a week),
- participating in any other physical activity program neither at school, nor after school,
- consent agreement of adolescents for participation,
- consent agreement of the parents/legal guardians for the participation of their children.

The exclusion criteria for the studied group were as follows:

- any data missing,
- diagnosed disabilities in cognitive or motor functions,
- pacemakers and other stimulators (forefending against bioelectrical impedance measurement),
- diagnosed with epilepsy (forefending against bioelectrical impedance measurement).

The inclusion criteria for the control group were as follows:

- Caucasian,
- adolescents aged 12–13,
- participating in the *Athletics for All* program neither currently, nor in the past,
- participating in any other physical activity program neither at school, nor after school,
- consent agreement of adolescents for participation,
- consent agreement of the parents/legal guardians for the participation of their children.

The exclusion criteria for the control group were as follows:

- any data missing,
- diagnosed disabilities in cognitive or motor functions,
- pacemakers and other stimulators (forefending against bioelectrical impedance measurement),
- diagnosed with epilepsy (forefending against bioelectrical impedance measurement).

The group of Caucasian adolescents was chosen as typical for the Polish population, i.e., a homogenous Caucasian one [27]; similar to a number of studies conducted for Polish population, such an inclusion criterion was applied [28,29].

In spite of the fact that in the systematic review of Bacil et al. [30] there is an association between biological maturation and physical activity, the maturation of the participating girls was not assessed to reduce the stress and embarrassment of adolescents, as well as to respect privacy and confidentiality [31]. It also resulted from the fact that an interaction of fat mass with maturation is often not observed [32].

**Figure 2:**
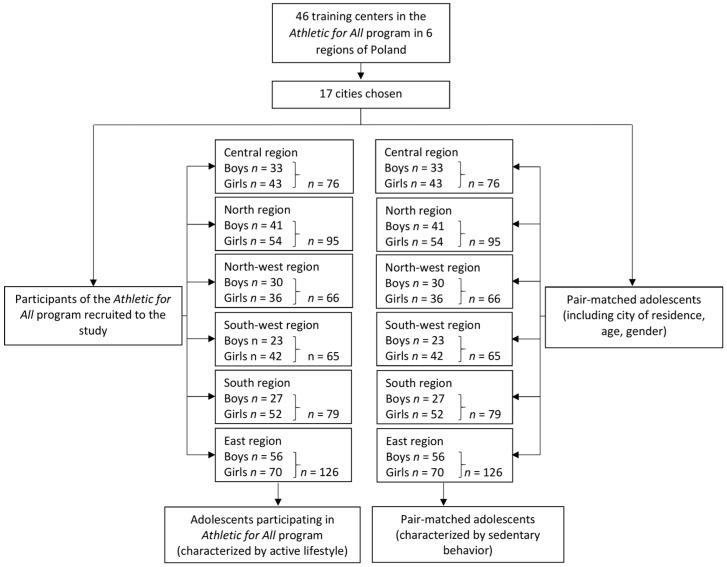
The geographical breakdown of the participants of the #goathletics Study.

At the end of the procedure, there were a total of 1014 adolescents (507 individuals representative of the *Athletics for All* program, including 210 boys and 297 girls, and 507 pair-matched individuals not participating in any physical activity program including 210 boys and 297 girls) recruited for the study. The geographical distribution of the participants is presented in Figure 2.

#### 2.3.2. Assessment of the Body Mass, Height, and BMI of the Study Participants

The body mass and height were measured by a professional dietitian, according to the widely accepted and applied rules [33], using a calibrated weighing scale (accuracy ± 0.1 kg) and a stadiometer (accuracy ± 0.5 cm). The BMI was calculated based on the Quetelet equation (body mass (kg)/height^2^ (m^2^)).

Following measurements, the obtained values were assessed on the basis of the Polish growth reference values. The body mass percentile, the height percentile and the BMI percentile of each participant were specified by gender and age [34] using the OLAF software [35].

Additionally, the BMI was interpreted according to the growth reference cutoffs for school-aged children and adolescents of the WHO [36], which are commonly applied [37]. BMI values were interpreted as follows: malnutrition—BMI < 5th percentile; normal weight—BMI Є < 5th–85th percentile); overweight—BMI Є < 85th–95th percentile); or obesity—BMI ≥ 95th percentile. The BMI percentile was assessed by gender and age on the basis of the Polish growth reference values [34].

The BMI percentile of the studied group stratified by region is presented in Table 1.

#### 2.3.3. Assessment of the WC and Waist-to-Height Ratio (WHtR) of the Study Participants

WC was measured by a professional dietitian, using a nonelastic flexible measuring tape (accuracy ± 0.5 cm). According to the widely accepted and applied rules, which were defined by the WHO [38] and International Diabetes Federation (IDF) [39], the mid-abdominal WC was measured in the horizontal plane midway between the lowest ribs and the iliac crest [40]. Following the measurement, the obtained values were assessed on the basis of the Polish growth reference values [41]. The individual WC values were compared with the 90th percentile value by gender and age, and the WC values were interpreted as follows: abdominal fat distribution—WC ≥ 90th percentile; or no abdominal fat distribution—WC < 90th percentile [42].

On the basis of the previously obtained measurements of body height and WC, the WHtR was calculated by dividing WC by height (waist (cm)/height(cm)) [43]. The WHtR values were interpreted as follows: central fatness—WHtR > 0.500; or no central fatness—WHtR ≤ 0.500 [44].

#### 2.3.4. Assessment of the Body Composition of the Study Participants

The body composition was assessed using bioelectrical impedance analysis. The measurements were taken by a professional dietitian using BIA 101/ASE (Akern Srl, Firenze, Italy). According to the commonly-applied rules, the measurements were obtained in the morning, in the fasting state (the last meal was to be consumed and the last beverage was to be drunk at least 8 h before the measurement), after a day when no excessive physical activity was performed; that is, no training was conducted on the previous day [45]. The measurements were taken after removing shoes and jewelry, and when the participants were in light underwear or sportswear with no metal elements.

The participants were asked to lie down on a two-layer polyurethane foam matte with no metal or conductive elements, and after 5 min of resting, measurements were recorded in a supine recumbent position according to the recommendations [46]. Before taking measurements, the dorsal surface of the right hand and right feet with no skin lesions were rubbed using medical disinfection cotton pads. The surface was allowed to dry, and two standard Ag-AgCl rectangular electrodes (Pro-Tab, PT 2334, Bio Protech, contact area higher than 4 cm^2^) were placed (with distance between the electrodes being at least 5 cm) for taking measurement in a tetrapolar electrode configuration. The measurements were conducted with arms separated from the trunk by about 30° and with legs separated by about 45° [47].

During the measurement, the data of resistance and reactance at a frequency of 50 kHz were recorded, while they remained stable. Following measurement, the Bodygram 1.31 software (Akern Srl, Firenze, Italy) and its equations were applied to calculate body cell mass (content, % of body mass, and body cell mass index), fat-free mass (content and % of body mass), fat mass (content and % of body mass), total body water (content, % of body mass, and Na/K ratio), extracellular water (content and % of water content), intracellular water (content and % of water content), and muscle mass (content and % of body mass).

### 2.4. Statistical Analysis

The obtained data were analyzed using Shapiro-Wilk test to assess the distribution. Afterwards, due to nonparametric distributions, the sub-groups were compared using Mann-Whitney U test (for nonparametric distributions of continuous variables) or *t*-Student test (for parametric distributions of continuous variables). The categorical variables were compared using a chi^2^ test. A level of significance of *p* ≤ 0.05 was accepted. The statistical analysis was conducted using Statistica, version 8.0 (Statsoft Inc., Tulsa, OK, USA) and Statgraphics Plus for Windows 4.0 (Statgraphics Technologies Inc., The Plains, VA, USA).

## 3. Results

### 3.1. Basic Anthropometric Characteristics

The basic anthropometric characteristics of the boys in the study group participating in a national athletics program and boys in the pair-matched control group are presented in Table 2. It can be noted that both body mass and BMI percentile differed significantly between the groups; for boys participating in a national athletics program, lower values of body mass and BMI percentile were observed compared to the control group of boys. Similarly, lower values of WC and WHtR were observed for the participants of national athletics program compared to the control group of boys.

The basic anthropometric characteristics of the girls in the study group participating in a national athletics program and girls in the pair-matched control group are presented in Table 3. It can be noted that both body mass and BMI percentile differed significantly between the groups; for girls participating in a national athletics program, lower values of body mass and BMI percentile and higher values of body height percentile were observed compared to the control group of girls. Similarly, lower values of WC and WHtR were observed for the participants of national athletics program compared to the control group of girls.

### 3.2. Assessment of Body Mass and Abdominal Fat Distribution

The body mass and abdominal fat distribution of the boys in the study group participating in a national athletics program and boys in the pair-matched control group are presented in Table 4. It can be noted that among the group of boys participating in a national athletics program, there was a significantly higher proportion of individuals with normal body mass and a significantly lower proportion of individuals with an excessive body mass (7.6% vs. 27.6%) compared to the control group of boys. Similarly, with respect to WC and WHtR, a higher frequency of abdominal fat distribution/central fatness was found in the control group of boys compared to the participants of national athletics program.

The body mass and abdominal fat distribution of the girls in the study group participating in a national athletics program and girls in the pair-matched control group are presented in Table 5. It can be noted that among the group of girls participating in a national athletics program, there was a significantly higher proportion of individuals with normal body mass and a significantly lower proportion of individuals with an excessive body mass (10.1% vs. 26.9%) compared to the control group of girls. Similarly, with respect to WC and WHtR, a higher frequency of abdominal fat distribution/central fatness was found in the control group of girls compared to the participants of national athletics program.

### 3.3. Body Composition

The body composition of the boys in the study group participating in a national athletics program and boys in the pair-matched control group are presented in Table 6. It can be noted that significantly higher values of body cell mass share, fat-free mass share, total body water share, extracellular water share, and muscle mass share were observed in the boys participating in a national athletics program compared to the control group of boys. Simultaneously, significantly lower values of fat mass share and intracellular water share were found in the participants of national athletics program than the control group of boys.

The body composition of the girls in the study group participating in a national athletics program and girls in the pair-matched control group are presented in Table 7. It can be noted that significantly higher values of body cell mass share, fat-free mass share, total body water share, extracellular water share, and muscle mass share were observed in the girls participating in a national athletics program compared to the control group of girls. Simultaneously, significantly lower values of fat mass share and intracellular water share were found in the participants of national athletics program than the control group of girls.

## 4. Discussion

On the basis of the Polish study conducted in a nationally representative random sample of involving 17,000 children and adolescents, it was stated that the prevalence of excessive body mass for the age group of 7–12 years is, depending on the applied definitions, 21.7–30.4% and 18.4–23.2% for boys and girls, respectively, as well as for the age group of 13–18 years—14.6–19.4% and 10.3–13.0% for boys and girls, respectively [48]. In the recent Polish study of Wadolowska et al. [49] conducted in the group of over 1500 adolescents aged 11–13, as a part of the ABC of Healthy Eating Project [50], the prevalence of excessive body mass was over 20%, while for 12% of the group, obesity was diagnosed.

In spite of the fact that the prevalence of excessive body mass in Polish adolescents is lower than in a number of European countries, the alarming trend of increase is indicated, as for the age group of 15 years, since from 2001 to 2014, the share of overweight and obese youth more than doubled [51]. Taking into account Polish statistics, the WHO [52] indicated the necessary actions that should be the key components of effective policy to be implemented in Poland in order to change the trend. Among other practical actions, there are those associated with the necessary physical activity interventions, in order to increase both school-based physical activity and extracurricular physical activity [50].

According to the WHO, it is recommend that children and adolescents aged 5–17 years should accumulate at least 60 min of moderate- to vigorous-intensity physical activity daily, while a higher amounts provide additional health benefits [53]. Similarly, the Centers for Disease Control and Prevention (CDC) of the United States of America, recommends for children and adolescents aged 6–17, 60 min or more of moderate-to-vigorous physical activity each day [54]. The Ministry of Health of the Republic of Poland also recommends at least 60 min of physical activity daily [55]. However, the prevalence of reaching the recommendations of the WHO regarding physical activity level for Polish adolescents aged 11–17 years is 28.5% for boys and 15.2% for girls [56].

In the conducted own study, it was not surprising to observe beneficial anthropometric measurements and body composition in the group of adolescents participating in a national athletics program in comparison with the pair-matched control group. However, the finding that all the observed parameters were better in the study group may be considered important, as it confirms the comprehensive positive effect of the after-school physical activity program assessed in this study. As the pair-matched samples of adolescents participating and not participating in a national athletics program were compared, while the gender and regional segmentation did not differ between sub-groups, the results may be interpreted as valid.

The present study showed that the recommended level of physical activity influenced not only BMI but also adiposity and the general body composition of the participants. For all the indicated parameters, physical activity was beneficial, which may confirm the positive influence of the national after-school athletics program. However, it must be emphasized that in some studies, even if after a physical activity intervention, some assessed parameters are improved, no success in adiposity reduction was observed [57].

The extracurricular program, assessed in the own study, was conducted in a school setting, included three sessions a week, and ensured regular participation, which made it most effective [18]. In particular, mandatory regular participation may have increased the effectiveness of the program, as it forced the participants to attend all the training sessions. However, this was possible only because the program was free of charge and regular participation was a condition for participation in program. Due to the fact that the parental socioeconomic status may be associated with the body mass of children [58], such actions that allow all children to participate in the physical activity program, independently from the economic status of family, may be especially effective. It results from the necessary actions that must be addressed excessive body mass pediatric population, that in the systematic review and meta-analysis of García-Hermoso et al. [59] were stated to be effective for obese ones.

While a new concepts of the educational programs are regularly analyzed [60,61], it is indicated that they must provide not only the effective results in terms of health promotion, but also be attractive enough to be associated with compliance, that being the necessary element for obtaining the required level of physical activity [62]. The unique strategy that was applied in the present study was associated with the indirect nutritional education via trainers, which may have contributed the obtained results.

Moreover, the fact that the program was an extracurricular one, but conducted in a school setting, may have influenced the observed results. No school-based obligation was applied, so it may be supposed that the participating adolescents were making their own decision to participate, which may have influenced their compliance, as, in general, such an association may be observed [63].

An additional advantage of the program was that it involved professional sport activities, but within the capacity of participants, as the program was organized by Polish Athletic Association and supported by the Ministry of Sport and Tourism. In spite of the fact that it aimed just to increase physical activity in a group of children and adolescents, by conducting regular athletics training, it also allowed adolescents to pursue sport as a future career by providing them with opportunities to meet professional trainers and even star athletes. Thus, the program was very attractive for participants, and it may be supposed that they were willing to participate.

The conducted program may be suggested to be effective after one year of intervention, but an important limitation of this study was that it was conducted in Poland and has not been verified in other countries thus far. Moreover, due to the fact that no baseline data were gathered, the progress attained by participants may not be assessed, and therefore, further studies are needed. However, the one-year intervention and the fact that the study was conducted in a randomly chosen group of adolescents participating in a real life program may allow us to conclude that the applied approach should be verified in other populations.

Such effective interventions, as implemented in Poland, may be crucial, as the excessive body mass and low physical activity levels observed in childhood and following through into adolescence [64] may continue in adulthood; therefore, reversing this situation is necessary to reduce the risks to general health [65].

## 5. Conclusions

The extracurricular physical activity program, conducted in school settings, including three sessions a week and ensuring regular participation, was highly effective for reduction of body mass, adiposity and improving body composition of adolescents after one year of intervention. To make any physical activity program effective, attractive training sessions and other elements must be planned, so that participants may be willing to participate.

## Figures and Tables

**Table 1:** The BMI percentile of adolescents participating in a national athletics program and pair-matched control group of adolescents stratified by region.

Region	Individuals Participating in a National Athletics Program		Individuals Non-Participating in a National Athletics Program	
	Mean ± SD	Median (Minimum–Maximum)	Mean ± SD	Median (Minimum–Maximum)
Central region (*n* = 76)	45.8 ± 24.2	44.0 * (1.0–94.0)	59.0 ± 29.5	67.5 * (3.0–99.9)
North region (*n* = 95)	52.9 ± 25.8	53.0 (1.0–99.0)	60.5 ± 28.0	63.0 * (1.0–99.0)
North-west region (*n* = 66)	48.1 ± 26.2	49.5 * (3.0–93.0)	57.8 ± 28.7	65.0 * (1.0–99.0)
South-west region (*n* = 65)	48.7 ± 28.1	52.0 * (2.0–97.0)	63.2 ± 28.6	62.0 * (4.0–99.0)
South region (*n* = 79)	43.7 ± 27.0	40.0 * (1.0–99.9)	63.2 ± 30.5	70.0 * (0.1–99.0)
East region (*n* = 126)	47.7 ± 25.7	47.5 * (1.0–98.0)	51.6 ± 28.8	49.5 * (1.0–98.0)

* Nonparametric distribution (verified using Shapiro-Wilk test; *p* ≤ 0.05).

**Table 2:** The basic anthropometric characteristics of group of boys participating in a national athletics program and pair-matched control group of boys.

The Assessed Parameters	Boys Participating in a National Athletics Program (*n* = 210)		Boys Non-Participating in a National Athletics Program (*n* = 210)		*p*-Value **
	Mean ± SD	Median (Minimum–Maximum)	Mean ± SD	Median (Minimum–Maximum)	
Body mass (kg)	47.18 ± 10.96	46.00 * (25.70–120.00)	50.16 ± 13.84	48.70 * (26.50–144.00)	0.0330
Body mass percentile (-)	50.71 ± 26.89	51.00 * (2.00–99.90)	57.40 ± 28.70	59.00 * (1.00–99.0)	0.0084
Body height (cm)	158.69 ± 10.27	157.50 (133.20–187.00)	157.72 ± 9.68	156.65 * (137.00–180.00)	0.3301
Body height percentile (-)	57.45 ± 29.59	59.00 * (1.00–99.90)	54.86 ± 29.26	54.00 * (1.00–99.00)	0.4268
BMI (kg/m^2^)	18.59 ± 2.80	18.10 * (14.00–34.30)	19.80 ± 3.76	19.20 * (14.00–35.50)	0.0003
BMI percentile (-)	47.06 ± 25.83	45.50 * (2.00–99.00)	57.13 ± 29.70	60.00 * (1.00–99.00)	0.0001
WC (cm)	68.85 ± 8.59	68.00 * (30.50–111.00)	73.28 ± 12.03	72.00 * (51.00–140.00)	0.0002
WHtR (-)	0.43 ± 0.05	0.42 * (0.20–0.68)	0.46 ± 0.07	0.45 * (0.32–0.78)	<0.0001

BMI—Body Mass Index; WC—Waist Circumference; WHtR—Waist-to-Height Ratio; * nonparametric distribution (verified using Shapiro-Wilk test; *p* ≤ 0.05); ** compared using *t*-Student test (for parametric distribution) and Mann-Whitney U test (for nonparametric distribution).

**Table 3:** The basic anthropometric characteristics of group of girls participating in a national athletics program and pair-matched control group of girls.

The Assessed Parameters	Girls Participating in a National Athletics Program (*n* = 297)		Girls Non-Participating in a National Athletics Program (*n* = 297)		*p*-Value **
	Mean ± SD	Median (Minimum–Maximum)	Mean ± SD	Median (Minimum–Maximum)	
Body mass (kg)	47.31 ± 9.66	47.30 * (25.50–87.90)	48.97 ± 11.23	48.20 * (23.50–88.00)	0.1146
Body mass percentile (-)	51.44 ± 26.90	53.00 * (0.10–99.90)	57.09 ± 29.18	58.00 * (0.10–99.90)	0.0117
Body height (cm)	157.99 ± 7.94	159.00 * (132.40–176.50)	155.54 ± 8.23	155.90 (132.50–175.70)	0.0001
Body height percentile (-)	55.91 ± 28.17	60.00 * (0.10–99.00)	49.29 ± 29.12	50.00 * (0.10–99.90)	0.0035
BMI (kg/m^2^)	18.62 ± 2.63	18.20 * (12.90–33.30)	20.06 ± 3.64	19.40 * (12.80–32.50)	<0.0001
BMI percentile (-)	48.60 ± 26.36	48.00 * (1.00–99.90)	59.42 ± 28.80	63.00 * (0.10–99.90)	<0.0001
WC (cm)	66.58 ± 7.06	66.00 * (48.00–102.00)	70.03 ± 9.66	69.00 * (50.90–106.00)	<0.0001
WHtR (-)	0.42 ± 0.04	0.42 * (0.33–0.63)	0.45 ± 0.06	0.44 * (0.32–0.64)	<0.0001

BMI—Body Mass Index; WC—Waist Circumference; WHtR—Waist-to-Height Ratio; * nonparametric distribution (verified using Shapiro-Wilk test; *p* ≤ 0.05); ** compared using *t*-Student test (for parametric distribution) and Mann-Whitney U test (for nonparametric distribution).

**Table 4:** The assessment of body mass and abdominal fat distribution in group of boys participating in a national athletics program and pair-matched control group of boys.

The Assessed Parameters		Boys Participating in a National Athletics Program (*n* = 210)	Boys Non-Participating in a National Athletics Program (*n* = 210)	*p*-Value *
BMI	Malnutrition	6 (2.86%)	10 (4.76%)	<0.0001
	Normal body mass	188 (89.52%)	142 (67.62%)	
	Overweight	12 (5.71%)	39 (18.57%)	
	Obesity	4 (1.90%)	19 (9.05%)	
WC	No abdominal fat distribution	183 (87.14%)	143 (68.10%)	<0.0001
	Abdominal fat distribution	27 (12.86%)	67 (31.90%)	
WHtR	No central fatness	190 (90.48%)	156 (74.29%)	<0.0001
	Central fatness	20 (9.52%)	54 (25.71%)	

BMI—Body Mass Index; WC—Waist Circumference; WHtR—Waist-to-Height Ratio; * compared using chi^2^ test.

**Table 5:** The assessment of body mass and abdominal fat distribution in group of girls participating in a national athletics program and pair-matched control group of girls.

The Assessed Parameters		Girls Participating in a National Athletics Program (*n* = 297)	Girls Non-Participating in a National Athletics Program (*n* = 297)	*p*-Value *
BMI	Malnutrition	10 (3.37%)	10 (3.37%)	<0.0001
	Normal body mass	257 (86.53%)	207 (69.70%)	
	Overweight	23 (7.74%)	47 (15.82%)	
	Obesity	7 (2.36%)	33 (11.10%)	
WC	No abdominal fat distribution	261 (87.88%)	205 (69.02%)	<0.0001
	Abdominal fat distribution	36 (12.12%)	92 (30.98%)	
WHtR	No central fatness	286 (96.30%)	243 (81.82%)	<0.0001
	Central fatness	11 (3.70%)	54 (18.18%)	

BMI—Body Mass Index; WC—Waist Circumference; WHtR—Waist-to-Height Ratio; * compared using chi^2^ test.

**Table 6:** The body composition in group of boys participating in a national athletics program and pair-matched control group of boys.

The Assessed Parameters	Boys Participating in a National Athletics Program (*n* = 210)		Boys Non-Participating in a National Athletics Program (*n* = 210)		*p*-Value **
	Mean ± SD	Median (Minimum–Maximum)	Mean ± SD	Median (Minimum–Maximum)	
Body cell mass (kg)	19.50 ± 4.58	19.10 * (8.90–35.70)	19.40 ± 4.91	18.55 * (10.90–43.50)	0.6416
Body cell mass (%)	52.05 ± 4.27	52.40 * (8.20–60.20)	51.83 ± 4.01	51.80 * (42.80–87.70)	0.0055
Body cell mass index (kg/m^2^)	7.67 ± 1.08	7.70 (4.50–10.80)	7.74 ± 1.36	7.65 * (5.40–15.50)	0.8992
Fat-free mass (kg)	37.17 ± 7.87	36.70 * (18.80–68.50)	37.42 ± 8.33	36.15 * (21.20–79.70)	0.9132
Fat-free mass (%)	79.15 ± 6.41	79.90 * (55.70–91.90)	75.64 ± 7.40	76.00 (55.30–99.30)	0.0000
Fat mass (kg)	10.05 ± 5.05	9.20 * (3.10–51.50)	12.96 ± 7.42	11.75 * (0.20–64.30)	<0.0001
Fat mass (%)	20.85 ± 6.41	20.10 * (8.10–44.30)	24.63 ± 8.24	24.25 * (0.70–77.10)	<0.0001
Total body water (kg)	30.62 ± 5.11	30.50 (14.50–49.60)	30.62 ± 5.29	30.00 * (13.90–56.30)	0.7692
Total body water (%)	65.55 ± 7.45	66.20 * (7.90–81.00)	62.84 ± 8.21	62.75 * (22.90–86.70)	0.0001
Na/K ratio (-)	1.13 ± 0.14	1.10 * (0.40–2.00)	1.10 ± 0.13	1.10 * (0.70–1.50)	0.0640
Extracellular water (kg)	12.76 ± 2.86	12.30 * (7.00–20.80)	12.60 ± 3.23	11.90 * (6.70–36.70)	0.3852
Extracellular water (%)	41.17 ± 3.73	41.40 * (10.30–50.00)	40.39 ± 3.19	40.00 * (32.20–60.50)	0.0021
Intracellular water (kg)	17.86 ± 2.45	17.80 * (7.50–29.80)	18.33 ± 2.69	18.00 * (11.60–34.00)	0.2453
Intracellular water (%)	58.68 ± 3.14	58.60 * (50.00–65.90)	59.66 ± 3.07	60.00 * (45.80–67.80)	0.0013
Muscle mass (kg)	24.08 ± 5.62	23.50 * (11.20–43.50)	23.91 ± 6.05	23.00 * (13.40–52.80)	0.5597
Muscle mass (%)	51.00 ± 6.19	51.35 * (5.90–64.90)	48.25 ± 6.15	48.35 * (32.70–83.90)	<0.0001

* Nonparametric distribution (verified using Shapiro-Wilk test; *p* ≤ 0.05); ** compared using *t*-Student test (for parametric distribution) and Mann-Whitney U test (for nonparametric distribution).

**Table 7:** The body composition in group of girls participating in a national athletics program and pair-matched control group of girls.

The Assessed Parameters	Girls Participating in a National Athletics Program (*n* = 297)		Girls Non-Participating in a National Athletics Program (*n* = 297)		*p*-Value **
	Mean ± SD	Median (Minimum–Maximum)	Mean ± SD	Median (Minimum–Maximum)	
Body cell mass (kg)	18.19 ± 3.60	18.30 (8.10–32.90)	17.85 ± 3.64	17.9 (8.90–28.10)	0.2604
Body cell mass (%)	51.88 ± 3.16	51.80 * (40.30–69.30)	51.08 ± 3.52	51.10 * (29.00–74.00)	0.0022
Body cell mass index (kg/m^2^)	7.29 ± 1.18	7.20 * (4.30–15.60)	7.58 ± 3.98	7.30 * (4.80–73.00)	0.3080
Fat-free mass (kg)	34.92 ± 5.93	35.10 (17.10–53.80)	34.92 ± 6.30	35.00 (17.90–51.20)	0.9936
Fat-free mass (%)	74.50 ± 5.74	74.60 * (56.00–99.70)	72.05 ± 6.27	71.70 (51.80–91.10)	<0.0001
Fat mass (kg)	12.63 ± 6.07	11.80 * (0.20–77.00)	14.14 ± 5.97	13.30 * (2.40–36.80)	0.0007
Fat mass (%)	25.60 ± 5.84	25.50 * (0.30–44.00)	27.97 ± 6.70	28.20 * (5.19–62.00)	<0.0001
Total body water (kg)	26.92 ± 3.37	27.10 * (13.10–37.10)	26.92 ± 3.57	26.80 * (13.70–36.00)	0.7667
Total body water (%)	58.12 ± 6.66	57.80 * (40.50–96.90)	56.22 ± 7.15	55.80 (39.50–73.60)	0.0009
Na/ K ratio (-)	1.07 ± 0.12	1.10 * (0.80–1.50)	1.06 ± 0.14	1.10 * (0.70–1.80)	0.2863
Extracellular water (kg)	11.57 ± 1.94	11.70 (6.30–17.50)	11.33 ± 1.94	11.30 (6.00–17.00)	0.1355
Extracellular water (%)	42.74 ± 2.46	43.00 * (34.40–49.70)	42.13 ± 2.31	42.30 * (33.50–48.30)	0.0003
Intracellular water (kg)	15.36 ± 1.50	15.40 * (6.80–19.80)	15.46 ± 1.74	15.50 * (7.40–20.00)	0.4845
Intracellular water (%)	57.26 ± 2.46	57.00 * (50.30–65.60)	57.89 ± 2.32	57.80 * (51.70–66.50)	0.0002
Muscle mass (kg)	22.42 ± 4.38	22.50 (10.10–40.00)	21.98 ± 4.39	22.00 (11.00–34.20)	0.2316
Muscle mass (%)	47.48 ± 5.45	47.50 * (5.40–83.80)	45.19 ± 5.37	45.20 * (23.90–81.10)	<0.0001

* Nonparametric distribution (verified using Shapiro-Wilk test; *p* ≤ 0.05); ** compared using *t*-Student test (for parametric distribution) and Mann-Whitney U test (for nonparametric distribution).

## Abbreviations

BMI	Body Mass Index
IDF	International Diabetes Federation
WC	Waist Circumference
WHO	World Health Organization
WHtR	Waist-to-Height Ratio
